# Silicate-, Magnesium Ion-, and Urea-Induced Prebiotic Phosphorylation of Uridine via Pyrophosphate; Revisiting the Hot Drying Water Pool Scenario

**DOI:** 10.3390/life10080122

**Published:** 2020-07-25

**Authors:** Maheen Gull, Arthur Omran, Tian Feng, Matthew A. Pasek

**Affiliations:** School of Geosciences, University of South Florida, Tampa, FL 33584, USA; ambermaheen@yahoo.com (M.G.); aomran@usf.edu (A.O.); tianfeng1@usf.edu (T.F.)

**Keywords:** phosphorylation, uridine mono-phosphates, prebiotic synthesis, origin of life, pyrophosphate, early earth

## Abstract

The availability of nucleotides on the early Earth is of great significance for the origin of a self-replicating system capable of undergoing evolution. We hereby report the successful phosphorylation reactions of the nucleoside uridine under heating in the “drying pool” prebiotic model at temperatures ranging from 60–75 °C, and by using pyrophosphate as a phosphorylation agent. Uridine monophosphates (UMP) such as uridine-5′-monophosphate (5′-UMP), 2′-UMP, and 3′-UMP, as well as cyclic 2′-3′-UMP, were identified by ^31^P-NMR. In addition to the above-mentioned products, a dimer of uridine-phosphate-uridine (U-P-U) was also observed. The reactions were promoted by white quartz sand, Mg^2+^, and by using urea as a condensation agent. The reactions also proceeded without this mixture; however, the yields increased remarkably with the presence of the above-mentioned materials. The results suggest that a hot/evaporating-drying pool of water containing organics, salts, and reactive phosphorus could be sufficient to form significant phosphate esters.

## 1. Introduction

During the process of origin of life on the Earth, it is believed that a self-replicating molecule must have been generated purely by chemical means and that this macromolecule could have been RNA, as suggested by the “RNA World” hypothesis [1,2,3,4]. The events leading to RNA, from the synthesis of nitrogenous bases and ribose, to the condensation of nucleosides and their eventual phosphorylation and self-assembly, are considered extremely significant in understanding the origin of RNA on the early Earth. It is suggested that the availability of phosphorus and the process of phosphorylation could have played a significant role in the process of chemical evolution [5,6,7]. The phosphorylation reaction is an endergonic thermodynamic reaction (requiring energy) and is overall favored at higher temperatures where it is coupled to water evaporation, but otherwise the lack of available energy makes the production of nucleotides for the emergence of RNA difficult [7,8].

Water is the most-accepted, prebiotically relevant solvent for reactions on the early Earth, and would likely have made the phosphorylation of nucleosides even more challenging since phosphorylation is favored by anhydrous conditions [5]. In order to resolve this difficulty, various anhydrous or semihydrous solvents have been promoted that demonstrate efficient plausible prebiotic syntheses of nucleotides [9,10,11,12,13], including the semiaqueous eutectic solvent consisting of urea, ammonium formate, and water, which has demonstrated both solubilization of phosphate and promotion of phosphorylation [14,15]. The previously suggested eutectic mixtures’ availability on the early Earth is questionable [12,13,14,15]. Furthermore, pools of formamide on the early Earth would have found it difficult to form spontaneously [16]. Formamide formation requires reaction between HCN and water, and the latter continues to convert formamide into ammonium formate [17,18]. Nevertheless, considering its universal availability and its ubiquity on the Earth’s surface, water is still considered to be the most plausible solvent for chemical evolution and prebiotic syntheses on the early Earth. Hence, the most plausible prebiotic scenarios should be water-based prebiotic reactions. Darwin’s “warm pond model” containing organics and phosphate substrates has been suggested to meet this need, which upon the removal of water by drying could keep the substrates intact and would be considered a site that would favor phosphorylation [16] and other condensation reactions [9,10,11,12,13,14,15].

However, presuming that water was the most likely solvent for prebiotic phosphorylation has its own challenges, as discussed above. The challenge of performing nucleoside phosphorylation reactions in water has been attempted to have been overcome by various ways such as (1) heating the solutions, containing orthophosphate (salts or minerals) and nucleosides, and a condensation agent (and/or minerals or clays as catalysts), to dryness [4,19,20,21,22,23,24,25,26,27,28,29]; (2) using reactive form of phosphorus, i.e., reduced oxidation state phosphorus species such as the meteorite mineral schreibersite [30] and other forms of reduced phosphorus species to induce phosphonylation [31]; and (3) using condensed polyphosphates such as trimetaphosphate (TMP), polyphosphate, triphosphate, and pyrophosphate. The condensed phosphates have shown promising results for the prebiotic phosphorylation of nucleosides [5]. This process is catalyzed by minerals and is promoted by condensation agents such as urea, and is capable of building RNA nucleotides in the presence of water as solvents [32,33,34,35,36,37]. Urea is considered to be a significant prebiotic condensation agent [5,6,7,14,15,22], which has also been shown to produce eutectic mixture with water to help facilitate the phosphorylation reactions [13,14,15]. Another key reactive phosphate compound called diamidophosphate (a condensed phosphate produced from TMP) has also shown excellence towards phosphorylation reactions of nucleosides under dry-paste conditions [38,39].

As the simplest polyphosphate, the phosphate dimer ion pyrophosphate (PPi (V); P_2_O_7_^4−^) is a noteworthy potentially prebiotic condensed phosphate. It is of great significance, as its proposed to be as a “plausible ancestor” of ATP molecule [40,41] and it is relatively stable [42]. It is likely to be prebiotically plausible, and its sources include hydrothermal systems [43,44,45] and routes via Fenton chemistry [46]. Potential prebiotic sources of pyrophosphate and the other condensed phosphates have also been discussed in detail by Keefe and Miller [47], who argued that though it might be prebiotically plausible, it is not terribly reactive. This is in contrast to cyclic TMP, of which no minerals bearing this anion are known [6].

Another reagent reported besides minerals and clays that could potentially facilitate phosphorylation is the Mg^2+^ ion [48], which is known to stabilize the diphosphate bonds in ATP molecule [7,48] due to formation of a stable complex between Mg^2+^ ion with oxygen and phosphorus of the ATP molecule [7,48]. The prebiotic significance of Mg^2+^ ion is aided by the findings that it can potentially promote the phosphorylation reactions of nucleosides approximately about 100 times more than other catalysts employed in the research study of Yamagata and others [49]. Moreover, it has been suggested that Mg^2+^ might have played a significant role in the phosphate geochemistry on the early Earth by assisting in the conversion of insoluble forms of phosphates such as apatites into more soluble minerals such as struvite, MgNH_4_PO_4_·6H_2_O [14].

Over the past few years, our group has concentrated on the prebiotic phosphorylation reactions of nucleosides, particularly by using the meteorite mineral schreibersite (or its synthetic analog Fe_3_P) [30] and by using nonaqueous solvents [12,13]. The reported yields of nucleotides of uridine and adenosine were relatively low when schreibersite (or Fe_3_P) was used as a phosphorylating agent (in the presence of urea and elevated temperatures) [30], while other methods afforded nucleotides in good yields but these reported methods employed nonaqueous solvents [12,13]. Our recent findings on utilizing silicates for efficient phosphorylation of glycerol encouraged us to attempt nucleoside phosphorylation by employing silicates as catalysts [13]. Silicates are considered to be the dominant group of minerals on the Earth’s crust. Schoonen and colleagues have discussed their significance and potential role in the origin of life [50]. Moreover, various silicates have been employed previously in different prebiotic synthesis reactions as discussed by Gull [7] (Please also see Tables 2 and 3 of [7]).

We hereby demonstrate phosphorylation reactions of aqueous solutions of the nucleoside uridine using pyrophosphate by heating at a temperature range of 60–75 °C for about 5 days, and in the presence of white SiO_2_ sand, urea, and Mg^2+^ ion under a simple prebiotic scenario of an evaporating/drying hot pool containing aqueous solutions of the reactants and other essential compounds such as urea and Mg^2+^ ion along with silicates. The temperature windows of 60–75 °C were chosen because of the following: (1) to mimic a hot drying/evaporating pool scenario; and (2) phosphorylation has been reported at these temperature windows, when water is the solvent (please see [5,6,7] for details); finally, water was chosen as a solvent due to its availability as the most realistic solvent on the early Earth.

## 2. Materials and Methods

Sodium pyrophosphate decahydrate (Na_4_P_2_O_7_·10H_2_O), urea, and magnesium sulphate heptahydrate (MgSO_4_·7H_2_O) were purchased from Fischer Scientific (Pittsburgh, PA, USA). Deuterium oxide (D_2_O) and standard 5′-UMP disodium salt hydrate were obtained from (Fair Lawn, NJ, USA), and uridine was obtained from Alfa Aesar (Tewksbury, MS, USA). White quartz sand (200–800 µm) grain size) also used in previously reported studies [13] was obtained from MP Biomedicals (Santa Ana, CA, USA). All chemicals utilized in the study were used as received, and deionized (DI) water was obtained by using a using a Barnstead (Dubuque, IA, USA) NANO pure^®^ Diamond Analytical combined reverse osmosis-deionization system [12,13].

### 2.1. Uridine Phosphorylation under ‘Warm-Pool Model’ Theme

In a typical experiment, 0.1 g Na_4_P_2_O_7_(about 0.22 mmoles), 0.5 g uridine (about 2 mmoles), 0.1 g urea (about 1.6 mmoles), 0.1 g magnesium sulphate (about 0.4 mmoles), and 0.3 g white sand (about 4.9 mmoles) were added to about 7–8 mL DI water and were stirred till a clear solution was obtained, aside from the white sand that settled at the bottom. The initial pH of the solution was around 6.5–7.5. The reaction vial was kept unsealed to allow the water to slowly evaporate under heating. The reactions were studied at temperature ranges over heating at 60–65 °C and 70–75 °C for about 5 days, and the individual effects of urea, white sand, Mg^2+^ as well as the effect of their mixtures were also observed. Table 1 provides the reaction conditions for each set of experiments.

### 2.2. ^31^P-NMR and Mass Spectrometry (MS) Analyses of Uridine Phosphorylation Reactions

^31^P-NMR spectra were obtained on unity INOVA spectrometer (161.84 MHz for ^31^P and 399.88254 MHz for ^1^H) (Varian, Palo Alto, CA, USA) equipped with a variable temperature controller and a Varian 5 mm Auto switchable probe with *Z*-axis gradient optimized for tuning of ^31^P and ^1^H [12,13,14,30]. The ^31^P chemical shift values were reported based on an external reference standard (neat 85% H_3_PO_4_ solution at room temperature, e.g., around 25 °C and δ ppm = 0.0). A ^31^P 45° flip angle pulse was used for proton-decoupled as well as nondecoupled spectra (90° ^31^P-pulse of 9.8 µs at 54 dB attenuation; the max power output is ~300 W). For proton-decoupled spectra, a composite pulse waltz decoupling sequence was applied, with a field strength of 2525 Hz during the acquisition time of 1 s and a relaxation time of 1 s. Dried samples were rehydrated with D_2_O and were centrifuged, and their ^31^P-NMR spectra were recorded. The signal was averaged from 264 transients.

In order to confirm the major product (5′-UMP), one of the reaction samples was spiked by adding standard 5′-UMP. Spiking experiments were performed by adding equal volume of reaction sample in two clean NMR tubes. The ^31^P-NMR spectrum of one NMR tube was taken as mentioned above. To the other NMR tube containing the reaction sample, about 0.015 g standard 5′-UMP was added, and the tube was shaken gently till all the standard was dissolved. The ^31^P-NMR of the tube “spiked” with the standard was also recorded. This step was done to confirm the presence of 5′-UMP.

Mass spectrometry (MS) analyses were done in negative ion mode on a 6130 Single Quadrupole Mass Spectrometer (Agilent, Santa Clara, CA, USA) attached to an Agilent 1200 HPLC by direct injection, and DI water was used as a solvent.

## 3. Results

**Figure 1:**
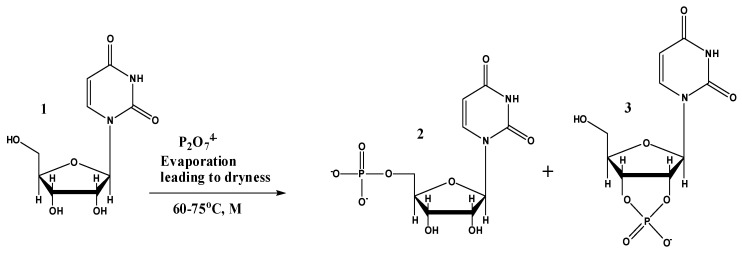
Main identified products of uridine phosphorylation reactions, where M = quartz sand, Mg^2+^ and urea and structure 1 shows uridine, structure 2 shows 5′-UMP, and structure 3 shows 2′,3′-cUMP. The reaction also afforded the formation of other phosphorylated products such as a dimer molecule U-P-U and 2′-UMP or 3′-UMP.

**Figure 2:**
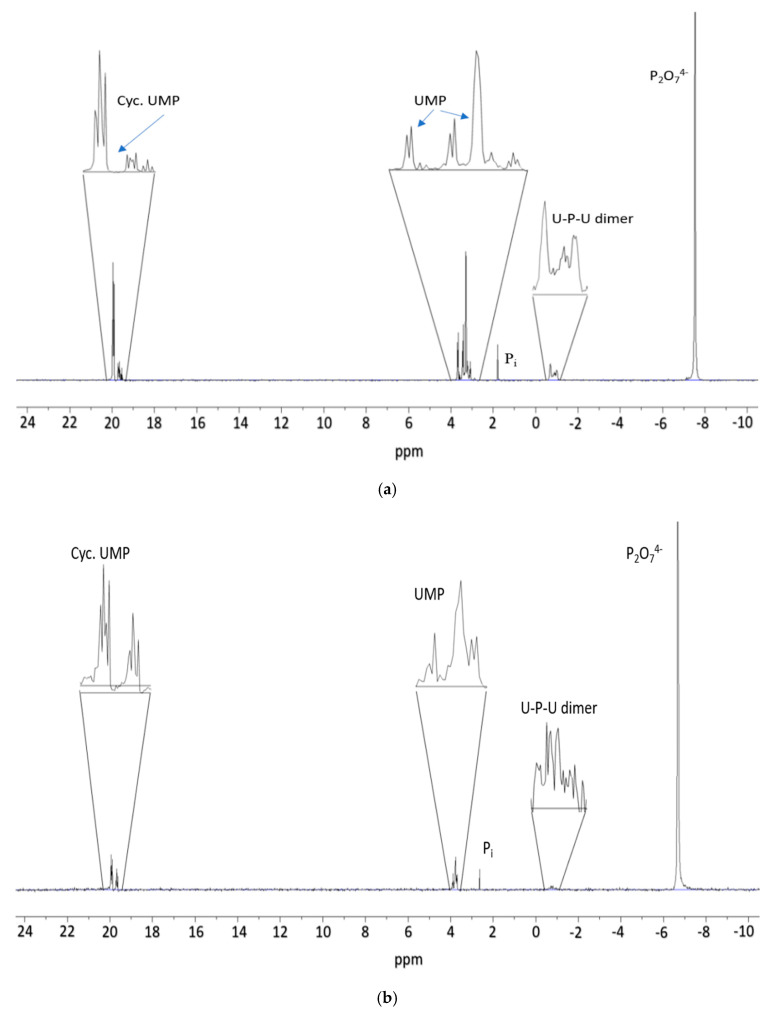

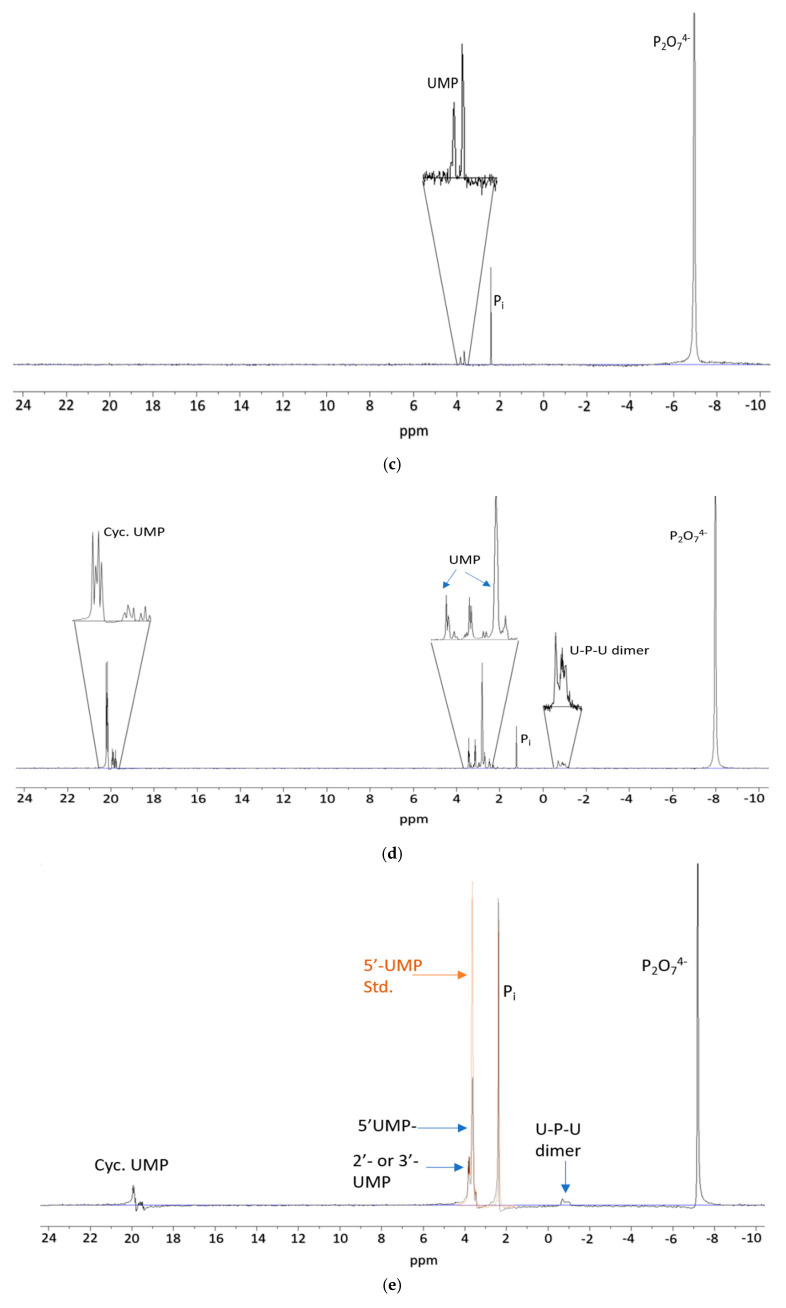
Phosphorylation reactions of uridine with pyrophosphate; (**a**) shows the proton coupled ^31^P-NMR spectrum of sample 1 with highest yields of the phosphorylated products. From right to left: around −8 ppm the peak shows pyrophosphate, the peak area between 0 and −2 ppm shows the U-P-U dimer species while around 1 ppm shows the orthophosphate (P_i_) and between 2–4 ppm the uridine monophosphates including 5′-UMP, 2′-UMP as well as 3′-UMP, respectively. The peak area around 19–20 ppm corresponds to cyc. UMP. This reaction was supported by urea, white sand, and Mg^2+^ ion and at 60–65 °C. (**b**) shows the proton coupled ^31^P-NMR spectrum of sample 8 carried out in the presence of Mg^2+^. Moving from right to left, dimer species U-P-U is still seen but less than in sample 1. (**c**) represents the proton coupled ^31^P-NMR spectrum of sample 10 “without” any additive material, and shows drastic decline in the yield of the phosphorylated products. (**d**) shows the proton-coupled ^31^P-NMR spectrum of sample 6 carried out at 70–75 °C and by using urea, sand, and Mg^2+^ ion mixture. Like sample 1 (**a**), sample 6 showed best possible yields of the phosphorylated products. (**e**) shows the results of spiking the sample with 5′-UMP to identify and match the major product. The black spectrum is the sample, whereas the orange spectrum in the figure represents the sample containing 5′-UMP standard.

In our experiments, evaporation led to dryness of the reaction solutions and formed phosphorylated products of uridine, including uridine monophosphates (2′-UMP and 3′-UMP) along with 5′-UMP. No diphosphates of uridine were observed based on the ^31^P-NMR analyses (Figure 1 and Figure 2). One of the most prominent findings was the formation of the dimer molecule of uridine-phosphate-uridine (where U-P-U could potentially represent following isomeric structures; U-5′-P-5′-U, U-2′-P-5′-U, U-3′-P-5′-U, U-5′-P-2′-U, U-5′-P-3′-U, U-2′-P-3′-U, U-3′-P-2′-U), which was identified by its location around −1 ppm of the ^31^P-NMR spectrum as well as by the multiplicity of the peaks when H-coupled. With the present methods not being specific to regiospecificity (e.g., MS merely shows the mass of the dimer, and ^31^P-NMR shows a multiplet at the correct chemical shift), we cannot resolve the specific bonds of these dimers. The molecular weight of the dimer molecule was also confirmed to be [C_18_N_4_O_14_PH_24_-H] at m/z 549 in the negative ion mode of MS. The other peaks confirmed were uridine-monophosphate (for 2′, and 3′ as well as 5′-UMP) [C_9_N_2_O_9_PH_13_-H] at m/z 323.04 and cyclic-uridine-monophosphate [C_9_H_11_N_2_O_8_P-H] at m/z 305.0, respectively. The ^31^P-NMR yields of products were calculated based on the peak integration methods as previously reported [12,13,30].

These ^31^P-NMR yields represent the proportional areas under the peaks as measured by NMR, and are normalized to 100% of the dissolved phosphorus species. In addition, since the number of scans were constant between each analysis, the signal to noise ratio of the pyrophosphate peaks could provide a semiquantitative estimate of the molarities of individual ions [51]. When the signal to noise ratio of the pyrophosphate peak in each experiment was referenced to the samples with no precipitation (samples 5 and 10), we found that samples 1, 2, 4, 6, 7, and 9 all had similar signal strengths compared to 5 and 10. This indicated that the total phosphorus content of the solutions was identical to the starting content, and thus had experienced no precipitation. However, in samples 3 and 8, the signal to noise ratio had decreased by about 60%, indicating loss of about an equivalent amount of phosphorus to precipitation. The speciation of phosphorus within this precipitate is unknown, but is likely inorganic phosphorus species (Pi and PPi) based on the respective solubility of these phosphorus ions [5]. Intriguingly, no precipitation occurred with the samples with MgSO_4_, sand, and urea, indicating that the Mg^2+^ is complexed and thus not promoting precipitation (echoing results of [17].

In the present work, the effects of urea, white sand, and Mg^2+^ ion were studied by employing each one of the proposed substances individually, as well as their combined mixtures. The results were also compared with the reactions without any of the aforementioned substances used. The best yields of the products (dissolved organophosphates) were obtained when the mixture of urea, white sand, and Mg^2+^ ion was employed with yields of organophosphates of up to 32.5%. Both temperature windows 60–65 °C and 70–75 °C produced almost produced similar results (Table 2). Individually, silicates, urea, and magnesium sulphate/reactants did not contribute appreciably to phosphorylation over the reagent/catalyst-free experiment. In the present research work, the key factor was found to be the catalyst mixture as both temperature windows yielded maximum yields only when this mixture was employed as a catalyst.

## 4. Discussion

A dimer species was produced in most of the samples with a relative yield ranging from 0.5 to 5% based on dissolved phosphorus. This dimer was formed in aqueous solution, and suggests polymerization reactions of uridine could occur in mild aqueous reaction conditions mimicking a hot evaporating pool of water. The environment most favorable to this reaction is within a hot evaporating pool of water. Such a site could potentially be a plausible site of RNA and other macromolecule syntheses and even more advanced condensation reactions, provided condensation agents, salts, and minerals were present. Cyclic UMP was also seen consistently, implying that the formation of the uridine monophosphates was quick and prolonged heating up to 5-days resulted in relatively higher yields of the cyclic derivative of UMP. The present work highlights the prebiotic synthesis of cyclic UMP (a phosphate diester) in mild aqueous environments. However, such phosphate diesters would require activation before these would contribute to oligomerize towards an RNA or pre-RNA worlds [52]. The presented work represents the synthesis of aforementioned phosphate diesters in simple prebiotically plausible conditions.

All samples, apart from samples 1 and 6 (Table 1) that contained all 3 reagents, produced organophosphates from about 5–10% of the added inorganic pyrophosphate (Table 2). Dimer was not detected in samples 5 and 10, implying that all three reagents, taken individually or together, enhanced the dimerization of urine phosphates. The samples containing the mixture of all three reagents (samples 1 and 6) led to the best relative yields, followed by those containing urea, white sand, or Mg^2+^ individually, which showed similar effects on the dimer formation.

In the presented reactions, most of the pyrophosphate remained unreacted (with only <30% release of P_i_ from pyrophosphate, i.e., hydrolysis of pyrophosphate to P_i_) and only samples 5 showed significant hydrolysis to P_i_ (64% orthophosphate, Table 2). Sample 5 showed major release of P_i,_ which could be perhaps attributed to the absence of additives or to a lower temperature window (60–65 °C), either of which may have favored the hydrolysis of pyrophosphate. It is important to mention here that high temperature and condensation agents thermodynamically favor generation of pyrophosphate from P_i,_ as discussed by Keefe and Miller, who suggested that at high temperatures in the presence of urea and other condensation agents the generation of pyrophosphate from phosphate is favored [47]. At present, we do not have a thorough explanation for this difference in hydrolysis yields. The reaction conditions of the experiments were mostly uniform, aside from added salts. Future work may provide an answer to this difference, which may have resulted from a combination of temperature, pH, or salt effects.

The phosphorylation of uridine was attempted under more plausible prebiotic conditions by (1) employing water as a solvent (in contrast to nonaqueous solvents such as UAFW (urea. Ammonium formate and water), formamide, and other deep eutectic solvents discussed earlier) and (2) employing catalysts that are likely to have been found on the early Earth. Indeed, as discussed previously, urea has been identified in Miller–Urey’s gas discharge tube experiments [53]; silicates are also undeniably prebiotically relevant, as suggested by Hazen and Sverjensky [54], and have been proposed as a significant component of the “prebiotic soup” [55], and Mg^2+^ has also been suggested to be prebiotically relevant [7,14,48,49].

A warm to mildly hot pool of water (60–70 °C) with dissolved nucleoside, coupled to a source of phosphate and minerals, clays, and simple condensation agents, could have given rise to the precursors of an RNA molecule through the formation of mono-phosphorylated nucleotides alongside nucleotide dimers. The present study also reveals that water is indeed challenging if it is considered as a solvent for phosphorylation. Furthermore, higher temperatures support phosphorylation reactions more, as suggested by Pasek [18], but this also becomes challenging because organics start decomposing rather quickly [56]. Phosphorylation based on water has been reported previously. However, the reactions reported were at relatively elevated temperatures and by using various other additives (please see Table 1 of [7]), and might not necessarily reflect a prebiotic “hot pool of water”.

We note here that this study is exploratory but demonstrates that phosphorylation of nucleosides and production of dimers is feasible at a temperature below the boiling point of water. This reaction necessitates an energetic phosphorus molecule (pyrophosphate), and a combination of at least two of the following: Mg^2+^, urea, and quartz sand. Mg^2+^ likely serves as a bridge between the two phosphates of the pyrophosphate, activating it [7,48] for phosphorylation. Urea is known to catalyze phosphorylation reactions [26,27,28,29] through the formation of a reactive phosphate intermediate that has yet to be identified by NMR. Quartz sand may catalyze the reaction by significantly increasing the surface area available for reaction, which may be crucial to the production of phosphorylated products [13]. It is apparent from our results that at least two of the compounds are necessary to promote efficient phosphorylation (> ~10% PPi). Future work will identify both the key reagent/catalyst necessary for effective phosphorylation, as well as investigate mechanistic detailed and more detailed identification of the products formed alongside their absolute yields.

Pyrophosphate is considered here to be a prebiotically plausible phosphorylation agent, because it is not only ubiquitous in hydrothermal environments [47,48] but is also a key product of the oxidation reactions of the reduced phosphorus compounds [51] and is found as a corroded product of meteorite mineral schreibersite [5,6,7,49]. It would be a significant finding if pyrophosphate generated from reduced oxidation state phosphorus compounds via Fenton chemistry [46] could prove to be a more reactive form of phosphate to form significantly higher yields of organophosphates under aqueous conditions.

## Figures and Tables

**Table 1:** Different reaction conditions given to each reaction sample.

Reaction Sets	Description
Set 1: To each sample, 0.1 g Na_4_P_2_O_7_ and 0.5 g uridine were added to about 7–8 mL DI water and dissolved to form a clear solution. The reaction temperatures were kept at a range of 60–65 °C for about 5 days. The remaining description, regarding the additional materials added to each of the sample in set 1, is as follows:	
Sample 1	0.1 g urea, 0.1 g magnesium sulphate, and 0.3 g white sand
Sample 2	0.1 g urea only
Sample 3	0.1 g magnesium sulphate only
Sample 4:	0.3 g white sand only
Sample 5	No additional material
Set 2: To each sample in this set, similar amounts of reactants were added as in set 2. However, the temperature window was 70–75 °C for about 5 days. The remaining description, regarding the additional materials added to each of the sample in set 2, is as follows:	
Sample 6	0.1 g urea, 0.1 g magnesium sulphate, and 0.3 g white sand
Sample 7	0.1 g urea only
Sample 8	0.1 g magnesium sulphate only
Sample 9	0.3 g white sand only
Sample 10	No additional material

**Table 2:** Yields^1^ (%) of various phosphates species detected in reaction samples of set 1.

Sample No.	P_2_O_7_^4−^	Pi	5′-UMP	2′- Or 3′-UMP	Cyc. UMP	Dimer (U-P-U)	Net Org. PO_4_
1	67	1	10.3	5.7	11	5	32
2	90	1	2.48	0.52	5	1	9
3	92.7	0.5	2.2	0.30	3.5	0.8	6.8
4	93.8	0.2	1	0.2	4.4	0.4	6
5	27	64	7.5	1	0.5	ND	9
6	66	1.5	10.8	4.2	14	3.5	32.5
7	91.2	1	1	ND	6	0.8	7.8
8	91.3	0.3	2.72	1.28	3.4	1	8.4
9	89.5	0.3	1.5	0.5	7	1.2	10.2
10	87.7	6.7	3.2	1.8	0.6	ND	5.6

^1^ The yields of the phosphorylated products as well as other inorganic phosphates were calculated on the basis of the total phosphorus dissolved and by the peak integration method, coupled to semiquantitative concentration estimation using signal to noise ratios, as previously reported [12,13], where Pi stands for orthophosphate; UMP stands for uridine-monophosphates; cyc. UMP means cyclic uridine monophosphates; and the dimer U-P-U represents uridine-phosphate-uridine, respectively; and ND means not detected. Please see Table 1 for the details relevant with the experimental conditions.
